# The Encephalophone: A Novel Musical Biofeedback Device using Conscious Control of Electroencephalogram (EEG)

**DOI:** 10.3389/fnhum.2017.00213

**Published:** 2017-04-26

**Authors:** Thomas A. Deuel, Juan Pampin, Jacob Sundstrom, Felix Darvas

**Affiliations:** ^1^Department of Neurology, Swedish Neuroscience InstituteSeattle, WA, USA; ^2^Center for Digital Arts and Experimental Media (DXARTS), University of WashingtonSeattle, WA, USA; ^3^School of Music, University of WashingtonSeattle, WA, USA; ^4^Department of Neurosurgery, University of WashingtonSeattle, WA, USA

**Keywords:** biofeedback, electroencephalogram, music, brain-computer interface, rehabilitation

## Abstract

A novel musical instrument and biofeedback device was created using electroencephalogram (EEG) posterior dominant rhythm (PDR) or mu rhythm to control a synthesized piano, which we call the Encephalophone. Alpha-frequency (8–12 Hz) signal power from PDR in the visual cortex or from mu rhythm in the motor cortex was used to create a power scale which was then converted into a musical scale, which could be manipulated by the individual in real time. Subjects could then generate different notes of the scale by activation (event-related synchronization) or de-activation (event-related desynchronization) of the PDR or mu rhythms in visual or motor cortex, respectively. Fifteen novice normal subjects were tested in their ability to hit target notes presented within a 5-min trial period. All 15 subjects were able to perform more accurately (average of 27.4 hits, 67.1% accuracy for visual cortex/PDR signaling; average of 20.6 hits, 57.1% accuracy for mu signaling) than a random note generation (19.03% accuracy). Moreover, PDR control was significantly more accurate than mu control. This shows that novice healthy individuals can control music with better accuracy than random, with no prior training on the device, and that PDR control is more accurate than mu control for these novices. Individuals with more years of musical training showed a moderate positive correlation with more PDR accuracy, but not mu accuracy. The Encephalophone may have potential applications both as a novel musical instrument without requiring movement, as well as a potential therapeutic biofeedback device for patients suffering from motor deficits (e.g., amyotrophic lateral sclerosis (ALS), brainstem stroke, traumatic amputation).

## Introduction

Since early in the history of the use of electroencephalogram (EEG) for measurement of electrical patterns of the human brain, efforts have been made to transform EEG electrical activity into sound. These efforts not only created diagnostic alternatives to purely visual feedback, but also opened up new possibilities for artistic expression, and created possibilities for therapeutic biofeedback.

The earliest example of converting EEG signal to sound appears in the literature shortly after the invention of the EEG. Adrian and Matthews (1934), replicating the earliest EEG descriptions of the posterior dominant rhythm (PDR; “the Berger rhythm”) by Berger (1929), monitored their own EEG with sound (Adrian and Matthews, 1934). Conversion of EEG signals to not just sound, but musical modalities, followed later: in 1965, the composer and experimental musician Lucier (1965) created a performance involving control of percussion instruments via strength of EEG PDR, with the encouragement and participation of composer John Cage. However, they experienced some difficulty in achieving good control, and to overcome this employed a second performer manually adjusting the gain from the EEG output (Rosenboom, 1975).

Following in Lucier’s pathway 5 years later, David Rosenboom in 1970 created a performance piece called “Ecology of the Skin” for Automation House in New York, NY, USA. This involved using EEG signal from 10 participants processed through individualized electronic circuits to generate visual and auditory performance (Rosenboom, 1975). More recently, Brouse et al. (2006), created EEG waveform spectral analysis in multiple frequency bands to passively control sound and music, in a project for the eNTERFACE summer workshop.

Eduardo Miranda at the Interdisciplinary Centre for Computer Music Research (ICCMR) at Plymouth University, UK was part of that summer workshop project, and has gone on to contribute significantly in this area of generating music from EEG signal. In 2008, he used the changing patterns of alpha and beta frequency rhythms in EEG to act as a switch between different musical styles (Miranda and Soucaret, 2008), and later used subject visual gaze direction to allow visual evoked potentials of EEG to control various musical parameters (Miranda et al., 2011). More recently, Miranda et al. (2011) used a statistical analysis of subjective emotions and EEG in an attempt to create an emotion sensor to subconsciously allow users to select music which they associate with more subjectively positive emotions (Eaton et al., 2014). Similarly, Makeig et al. (2011) used EEG and non-EEG signal (scalp muscle and eye movement) from one subject to drive the use of subjective emotions to control a series of musical intervals.

Pham et al. (2005) used slow cortical potentials of EEG to drive control of either ascending or descending pre-set pitch sequences; they used both auditory feedback and visual feedback. While they used tone sequences for feedback, the feedback did not represent a musical context. Using this protocol, they observed significantly better results for visual than auditory feedback. Hinterberger and Baier (2005) used six different frequency bands of EEG signal to drive multiple sound parameters in 10 subjects, some of whom were able to control some sound patterns significantly when prompted with a visual stimulus.

Some of the devices described above which use conscious control can be considered Brain Computer Interfaces (BCIs; Wolpaw and Wolpaw, 2012). BCI research has progressed significantly in advancing towards the goal of using non-invasive EEG scalp electrodes to generate a direct interface from brain signal to a computer to control such actions as moving a cursor on a screen or a word speller (Sellers et al., 2014), driven by signals such as alpha frequency event-related desynchronizations and synchronizations (Roberts et al., 1999; Pfurtscheller et al., 2000). Here, we sought to use the well-described methods of using PDR and motor imagery EEG real-time control to create a new scalar musical instrument, and to measure its accuracy for novices.

In this article, we describe the creation of the Encephalophone, a musical instrument and biofeedback device that uses visual cortex PDR or motor cortex mu rhythm (mu) to consciously and volitionally control the generation of scalar music. PDR was used due to the simplicity of instructions for novices, as instructions involve opening and closing the eyes. Mu rhythm was used to generate an instrument that can be controlled without movement (Yuan and He, 2014) for potential applications with patients with motor disabilities. Alpha frequency control using both PDR and mu rhythms has been well described in the BCI literature (Roberts et al., 1999; Pfurtscheller et al., 2000) for non-musical control. We additionally describe experiments demonstrating accuracy for novice users which is significantly higher than random in controlling the instrument by conscious cognitive processes, and show that PDR control was significantly more accurate than mu control.

## Materials and Methods

### Ethics Statement

The written informed consent was obtained from each subject prior to testing, and subjects had the opportunity to withdraw from the study at any time. IRB approval (application #49770) as obtained from the Human Subjects Division of the University of Washington (Seattle, WA, USA).

### Recruitment of Subjects

The subjects were recruited from email and fliers to undergraduate and graduate students at a university setting. Inclusion criterion was healthy adults, and exclusion criteria was age less than 25 or greater than 65.

### EEG Signal Collection

**Figure 1:**
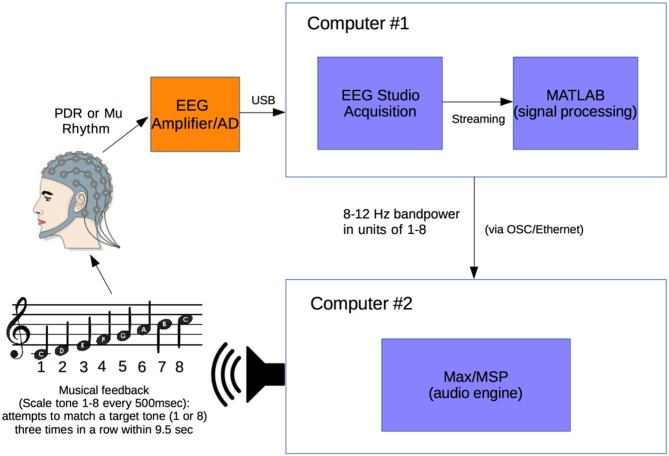
**Experimental setup**. Electroencephalogram (EEG) signal from subject wearing electrode cap is sent from Mitsar 201 EEG amplifier to Computer #1 where 8–12 Hz posterior dominant rhythm (PDR) or Mu power is converted to a value from 1 to 8. This value from 1 to 8 is sent via OSC to Computer #2 where it is converted to a musical piano tone in the key of C (seven tones of C major scale and octave, from C4 to C5). Subjects generating tones attempt to match them with a presented target tone.

Figure 1 illustrates the experimental setup (Figure 1). A Mitsar 201 EEG (Mitsar Co., Ltd., St. Petersburg, Russia; distributed by Nova Tech, Inc., Mesa, AZ, USA) and 19-channel ElectroCap electrode cap (Electro-Cap International Inc., Eaton, OH, USA) were used to collect EEG signal utilizing the International 10-20 system of electrode placement (American Electroencephalographic Society, 1994) from 15 healthy human volunteer subjects.

Subjects were positioned in a relaxed, reclining position with a headrest to minimize muscle artifacts, and were positioned facing away from computer screens and other equipment to eliminate any potential for visual feedback. EEG signal at a sampling rate of 500 Hz was initially processed in a HP Pavilion PC (Hewlett-Packard, Palo Alto, CA, USA) with Mitsar EEG Acquisition software, where filters were applied (100 Hz low-pass, 0.5 Hz high-pass, and 60 Hz notch filters). Raw EEG signal was visually verified by a physician clinical neurophysiologist for good signal quality and lack of artifacts. EEG data was then streamed in real time to Matlab (The MathWorks, Inc., Natick, MA, USA) via the Mitsar Matlab API.

Matlab scripts for real-time signal processing were created to apply a fourth order Butterworth filter at the 8–12 Hz band to generate an estimate of power for the PDR in visual cortex from occipital electrode O1, or motor cortex mu rhythm from electrode C3 (international 10-20 system) for right hand motor imagery, in real time. The delay in the system from EEG signal acquisition to Matlab processing was approximately 20 ms. The filter was applied to incoming segments of 500 ms of data. The bandpass filtered data was rectified and then averaged over the entire segment length to produce a single power estimate for every segment.

### Calibration Period

A calibration was created for each individual subject and each individual trial session of the Encephalophone. The 5 min long calibration period consisted of 20 15 s long alternating cued states (“on” or “off”). For visual cortex PDR, an auditory cue of “on” cued the eyes closed, awake state, and “off” cued the eyes open, awake state. For motor cortex mu rhythm, an auditory cue of “on” cued the awake, resting state and “off” cued the motor imagery (but not actual movement) state: subjects were instructed to imagine right hand grasping and opening at a rate of approximately 1 Hz as per prior motor imagery BCI methods of Neuper et al. (2006). This calibration period established the range of values of 8–12 Hz power for an individual subject and individual trial session in the different cued states, then divided these values into eight equal sized “bins”, or ranges of values, based on the calibration period alpha power histogram. After calibration, these eight possible values generate the 8 scale degrees of the C major musical scale including the octave (C4 to C5).

After the calibration period is used to calibrate the instrument to each individual, the device enters the free-running period, during which a value from 1 to 8 is generated every 500 ms in real-time from the desired 8–12 Hz frequency power (PDR or mu rhythm) of the user. Subjects were allowed brief (3 min) free-running practice with note generation before accuracy experiments.

This free-running stream of values from 1 to 8 in Matlab is sent at a rate of one value per 500 ms (120 bpm musical tempo for quarter notes) using Open Sound Control (OSC) along an Ethernet cable via a router to a second computer—an Apple MacBook Pro (Apple, Inc., Austin, TX, USA)—where it is received by Max/MSP music generation software (Cycling ’74, Walnut, CA, USA). The streaming values from 1 to 8 are used to generate the 8 scale degree notes in the C major musical scale with a synthesized piano tone (eight notes from C4 to C5).

### Accuracy Experiments

For note accuracy experiments, the subject is presented with a target note of either a high C (C5) or low C (C4). The subject generates one note every 500 ms and attempts to match the note (C4 or C5) or its nearest neighbor (D4 or B4) three times consecutively. If the note is successfully matched three times consecutively, a “hit” is scored and a reward chord (C major) is played, then a new target note is presented. If the subject does not hit the target note three times consecutively within 9.5 s (19 notes), a “miss” is scored and an error chord (tritone) is played, then a new target note is presented. This results in a chance probability of 19.03% to score a “hit” over the interval. A total of 300 s, or 5 min, is given for each trial, and the results recorded.

### Statistical Analysis

Statistical analysis was done in conjunction with consultation from the Department of Biostatistics, University of Washington, Seattle, WA, USA. Statistical analysis of *p*-values was performed using the binomial cumulative distribution for individual subjects. The significance of the difference between two means was calculated using the Generalized Linear Mixed Model (GLMM; Breslow and Clayton, 1993) for comparing the PDR condition to the Mu condition. We used the GLLM test because unlike other non-parametric tests (such as the Wilcoxon signed-rank test), the GLLM tests binary data, clustered data and tests the odds of a hit being significantly different from a miss (rather than the odds that the distributions are significantly different with the Wilcoxon signed-rank test). Statistical analysis of *p*-values for skewness was done using the student’s *t*-test with sample population standard deviation.

## Results

Fifteen healthy adult volunteer subjects were trained and tested for musical accuracy using the Encephalophone using both PDR control and motor mu rhythm control (basic subject demographics shown in Table 1). Subjects underwent a 5 min calibration period, followed by a brief (3 min) free-run practice period, then a 5 min accuracy trial for each of PDR and mu control. Results from these musical accuracy experiments were recorded for individual number of hits, trials and percent accuracy for each 5 min trial using PDR control and mu control (summary shown in Table 2).

**Table 1:** **Subject demographics**.

Subject #	Age	Gender	Years musical training
1	27	M	18
2	63	M	3
3	44	M	18
4	28	F	18
5	28	F	7
6	42	F	14
7	37	M	0
8	35	M	0
9	38	F	5
10	31	M	25
11	27	M	19
12	25	F	11
13	27	M	8
14	48	M	6
15	32	F	10
Average	35.5		10.80

**Table 2:** **Individual subject results from accuracy experiments**.

Subject #	PDR hits	PDR trials	PDR %	*p* value	Mu hits	Mu trials	Mu %	*p* value
1	16	35	45.7	7.41E-05	17	35	48.6	1.69E-05
2	23	36	63.9	5.69E-10	32	41	78.0	3.11E-17
3	33	39	84.6	6.57E-20	25	39	64.1	1.08E-10
4	23	40	57.5	1.28E-08	27	39	69.2	1.20E-12
5	30	41	73.2	8.22E-15	19	35	54.3	6.36E-07
6	52	55	94.5	6.34E-36	28	38	73.7	3.47E-14
7	30	38	78.9	1.40E-16	16	33	48.5	2.81E-05
8	14	32	43.8	3.20E-04	17	33	51.5	5.72E-06
9	12	31	38.7	2.79E-03	27	40	67.5	3.28E-12
10	52	55	94.5	6.34E-36	19	34	55.9	3.32E-07
11	34	41	82.9	7.95E-20	19	33	57.5	1.67E-07
12	19	33	57.6	1.67E-07	15	33	45.5	1.24E-04
13	24	27	88.9	1.71E-11	12	31	38.7	2.79E-03
14	25	41	61.0	5.65E-10	17	35	48.6	1.69E-05
15	23	37	62.2	1.32E-09	19	35	54.3	6.36E-07
Average	27.4	38.7	67.1		20.6	35.6	57.1	
Random			19.03				19.03	

*Number of hits, number of trials, and percent (%) accuracy with p values for both PDR control and mu control*.

**Figure 2:**
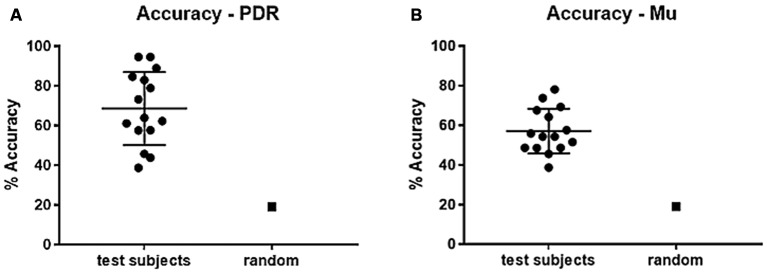
**Percent accuracy for PDR and Mu control**. Individual subjects were given 5 min to hit as many target notes as possible using either PDR control or Mu control. Scatter plots of results of all subjects were generated (bars represent mean and standard deviation), with random (chance) control, for each of: **(A)** Percent accuracy using PDR control (standard error ± 4.7). **(B)** Percent accuracy using mu control (standard error ± 3.0).

Subjects using PDR control had an average of 27.4 hits (standard deviation = 11.9, standard error ± 3.2) in an average of 38.7 trials, resulting in an average of 67.1% accuracy (Figure 2A, standard deviation = 17.42%, standard error ± 4.5%). Subjects using mu control had an average of 20.6 hits (standard deviation = 5.7, standard error ± 1.5) in an average of 35.6 trials, resulting in an average of 57.1% accuracy (Figure 2B, standard deviation = 11.2%, standard error ± 3.0%). Each individual subject scored significantly higher than random in accuracy for both PDR and mu control (Figure 2): *p* values ranged from 6.3 × 10^−36^ to 2.8 × 10^−3^. Additionally, PDR accuracies (average 67.1%) were significantly higher (*p* = 1.4 × 10^−4^) than Mu accuracies (average 57.1%).

**Figure 3:**
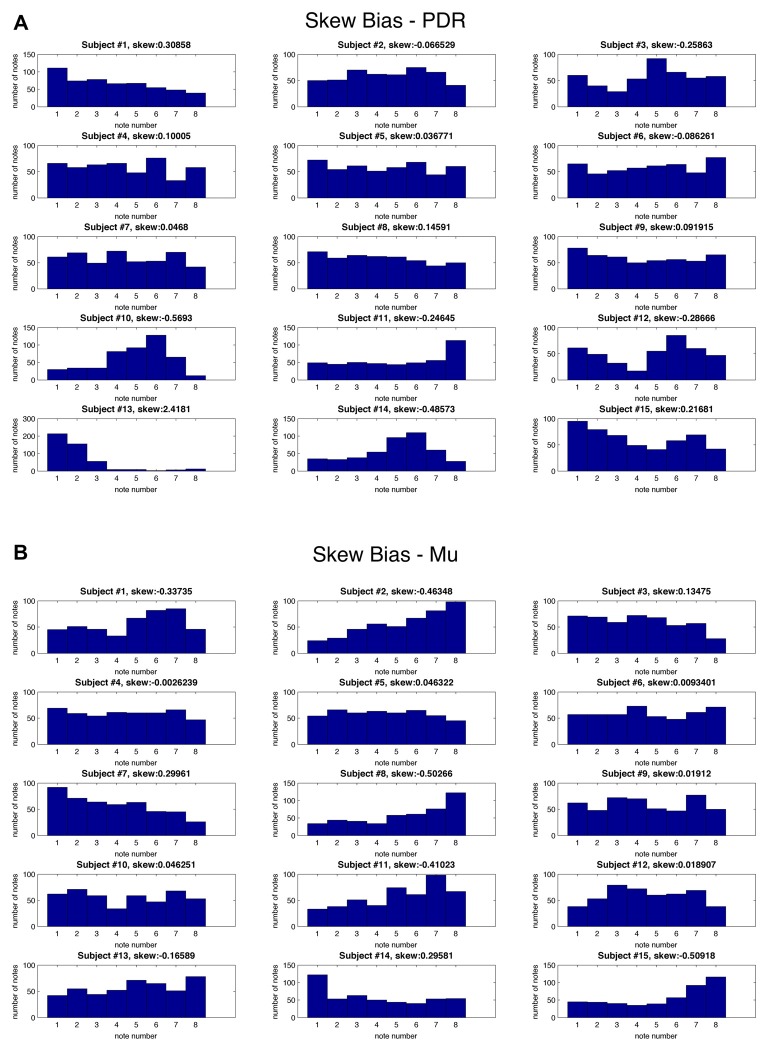
**Skew bias for individual notes for each subject**. Histograms for each individual subject showing frequency (*y* axis) of each of eight possible musical notes (*x* axis) as well as skewness values during testing for: **(A)** PDR control experiment and **(B)** Mu control experiment.

In order to assess for individual subject bias skewed towards particular notes, histograms of note generation during each 5 min testing session were created, and skewness calculated (Figure 3). If an individual exclusively generated a high or low note, for example, this bias would result in scoring a hit in 50% of note trials. Extreme skew bias could occur, for example, if no alpha frequency signal was recorded during calibration. Note that the task itself of hitting one of two notes at the extremes of the note range may generate skewness towards the extremes (notes 1 and 8). There was a range of skew bias, with the most significant skew bias towards low note generation (skewness +2.42, *p* = 2.1 × 10^−9^) with subject #13/PDR, and toward high note generation (skewness −0.57, *p* = 6.8 × 10^−3^) with subject #10/PDR. In these two most biased cases, if we look at their accuracy with the non-biased target note alone (i.e., throw out biased target note trials), they score 61.1% (*p* = 1.10 × 10^−6^), and 67% (*p* = 1.80 × 10^−8^) note matching accuracy, respectively, significantly above random chance (19.03%).

**Figure 4:**
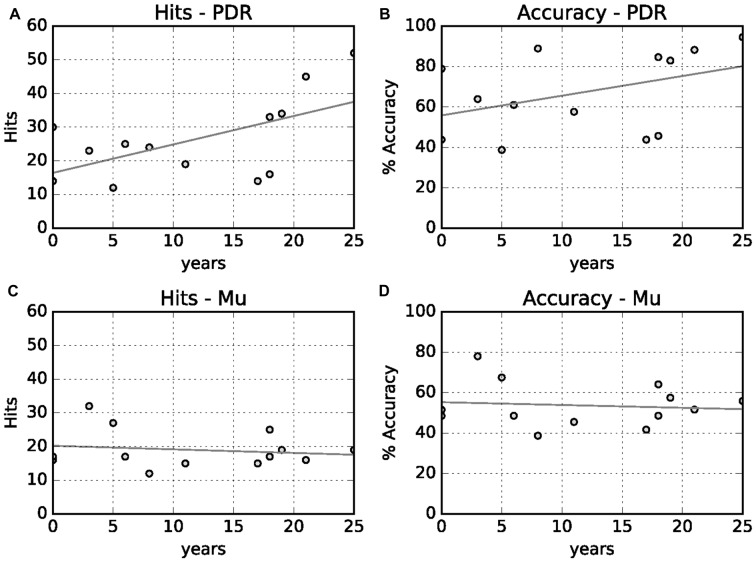
**Relationship between years of musical training with hits and accuracy. (A)** Years musical training vs. PDR hits (correlation value 0.58, *p* = 0.01). **(B)** Years musical training vs. PDR accuracy (correlation value 0.41, *p* = 0.06). **(C)** Years musical training vs. mu hits (correlation value 0.16, *p* = 0.28). **(D)** Years musical training vs. mu accuracy (correlation value −0.11, *p* = 0.35).

We also looked at the correlation between PDR hits and accuracy, and mu hits and accuracy, with years of musical training (Figure 4). There was a moderate positive relationship between increased PDR hits and accuracy (correlation values 0.58 and 0.41, respectively)—but not mu hits and accuracy (correlation values −0.16 and −0.11, respectively)—with increasing years of musical training.

## Discussion

This article describes the creation of the Encephalophone, a musical instrument and biofeedback device, which uses either PDR or mu rhythm EEG signal to control notes of a musical scale in real time. We describe testing 15 normal subjects novice to the device in experiments to test accuracy in hitting a target note, and our results show each subject scoring significantly higher than random, with the average score much higher than random for both PDR and mu control. Mu control average accuracy is comparable with that previously shown (56%) by studies using motor imagery mu control (Neuper et al., 2005). PDR control showed significantly higher accuracy than mu control for these novices, as might be expected given the more straightforward task of opening and closing eyes—as opposed to increasing and decreasing motor imagery. We additionally looked at skew bias for individual notes for each subject, and found most subjects without large skew bias. Even those subjects with larger biases were able to score hits at both ends of the note range. We additionally found a moderate positive correlation between years of musical training and PDR accuracy, but not mu accuracy.

These studies demonstrate that the Encephalophone allows novices to have some cognitive volitional control of generation of musical notes in real time, without movement. We believe these results and the creation of this device is of significant interest for several reasons. First, the Encephalophone represents a novel musical instrument that uses EEG control to create scalar music in real-time, and allows some basic accuracy that has been experimentally tested here. Second, given the known potential for significant improvement with training in mu-based BCI devices (Neuper et al., 2006), novices such as those tested here have the potential with continued training to significantly improve accuracy and facility with the instrument. The use of scalar musical tones—rather than non-musical sound or visual biofeedback—may confer a training advantage: the benefits of music for arousal motivation for both training and therapeutics have been shown (Bergstrom et al., 2014). Third, the use of a musical feedback-based EEG device with responsiveness noticeable to the user may hold promise for patients—such as those with locked-in syndrome—who are severely incapacitated and may be more likely to respond to auditory (and specifically musical) stimulus and feedback than to visual stimulus and feedback. This is particularly so for those who may have visual impairment (e.g., cortical blindness), and particularly to those who played music before their injury.

Previously, others have reported use of BCI to control not only visual output (e.g., cursor on a computer screen) but also sound, and reported better control and accuracy with visual rather than (non-musical) auditory feedback (Nijboer et al., 2008). However, Bergstrom et al. (2014) showed musical biofeedback to be better than either simple passive music listening or non-musical sonification biofeedback for control of physiological arousal state. Here we report reasonable control with virtually no training, using scalar musical tone feedback rather than non-musical auditory feedback. Thus we hope that with further training involving musical accompaniment between testing sessions, the musical context provided will greatly improve learning and accuracy of control. This will be tested in future experiments with serial training, as well as testing with more note target options (3 or 4 possible notes to match rather than 2).

This device is being used as a novel improvisational musical instrument in live performance, accompanied by small ensembles of musicians. Future development will include using multiple soloists performing with Encephalophones together, in a call and response improvisation, as well as performers improvising not only with musical scales, but also with timbre or chordal improvisation. Furthermore, work in computer music using conscious control of sound spatialization is being explored.

Diagnostically, the Encephalophone might prove useful in auditory monitoring of clinical EEG applications, such as auditory seizure detection, as described by Loui et al. (2014). Therapeutically, we also plan on using the Encephalophone in trials of cognitive rehabilitation and neurologic music therapy with patients with motor disabilities (but with at least one intact motor cortex). It has already been demonstrated that neurologic music therapy improves executive function in traumatic brain injury rehabilitation (Thaut et al., 2009). Other patients who might thus benefit would include patients suffering from amyotrophic lateral sclerosis (ALS), brainstem stroke, or traumatic amputation (such as war veterans). The ability to generate music using a portion of the brain that is no longer able to control motor movement of limbs may be beneficial for emotional and cognitive rehabilitation. Also, combining the Encephalophone with physical therapy may improve motor rehabilitation, and cortical “rewiring” of motor circuits may allow new motor output pathways for regaining some motor control.

## Author Contributions

TAD contributed the largest amount to all aspects of the work—conception of design of the Encephalophone, experimental design, acquisition of the data, analysis of the data, as well as drafting and editing the manuscript. JP contributed to all of this aspects of the work, but particularly experimental design, analysis of data and drafting and editing the manuscript. JS contributed to all of this aspects of the work, particularly in experimental stimulus design, acquisition of data, analysis of data and drafting and editing the manuscript. FD contributed the second largest amount to all aspects of the work, particularly in design of the Encephalophone, experimental design, analysis of the data, as well as drafting and editing the manuscript.

## Funding

This research received no outside funding. The authors received no specific funding for this work.

## Conflict of Interest Statement

The authors declare that the research was conducted in the absence of any commercial or financial relationships that could be construed as a potential conflict of interest.
